# Erroneous Central Venous Catheter Placement: Multidisciplinary Primary Surgical Repair of the Vertebral Artery

**DOI:** 10.7759/cureus.22933

**Published:** 2022-03-07

**Authors:** Patrick J Opperman, Jonathan R Thompson, Daniel L Surdell

**Affiliations:** 1 Neurosurgery, University of Nebraska Medical Center, Omaha, USA; 2 Vascular Surgery, University of Nebraska Medical Center, Omaha, USA; 3 Neurosurgery and Neurological Surgery, University of Nebraska Medical Center, Omaha, USA

**Keywords:** vascular surgery, neurosurgery, primary surgical repair, iatrogenic injury, central venous catheter, vertebral artery

## Abstract

Central venous catheters are a common practice in critical care medicine. These lines are of particular importance when a patient needs large volume resuscitation or medications that cannot be infused through a peripheral line. Even though central venous catheters are frequently utilized, they are associated with potentially significant risks that one must be aware of when attempting placement. The anatomy and pertinent complications are key for any healthcare professional to be aware of during this procedure. As such, vascular injury has been described in the literature, but vertebral artery injury and common repair techniques are less common. Primary repair of the second vertebral artery segment is infrequently detailed in the literature and this report describes pertinent case details and plan of action for identification and repair of iatrogenic vertebral artery injury following catheter placement.

## Introduction

The placement of central venous catheters (CVC) is a mainstay in medicine for administering large volume resuscitation and some subsets of medications, such as vasopressors. While this is a common procedure, catheter placement is not without complications. Several common complications include vascular injury, misplacement, and line-associated infection [1]. As we have seen firsthand, vascular complications are a significant possibility.

Given the vascular anatomy presented during this procedure, arterial and venous injuries are possible. Arterial injuries are uncommon, occurring in less than 1% of CVC placements [2]. When this occurs it can often be caught given the pulsatile nature and increased flow pressure, but this is made difficult in the severely ill patient in the intensive care unit (ICU) [3]. Arterial injury has its highest possibility in femoral line placement and lowest likelihood in subclavian placement [4]. The risk of arterial cannulation is approximately 3% with attempted internal jugular vein (IJV) catheterization compared to 0.5% with attempted subclavian catheterization [5].

Vertebral artery (VA) injury with CVC placement is a rare occurrence and literature review reveals primarily case reports detailing management. Rapid identification of VA injury can be difficult given its deep position in the neck, leading to delayed presentation with pseudoaneurysm compression of adjacent structures or symptoms of arteriovenous fistula formation being common presentations [6].

We describe a case of inadvertent vertebral artery injury that occurred during the attempted placement of an internal jugular CVC. We detail the multi-team approach for bony vertebral exposure by neurosurgery and primary open surgical repair of the vertebral artery by vascular surgery. 

## Case presentation

An adult male presented to an outside hospital with work-up revealing acute aortic endocarditis and methicillin-resistant Staphylococcus aureus bacteremia, in the setting of known intravenous drug use and polysubstance use disorder. He subsequently decompensated, requiring pressor support and intubation. The patient was initially treated in the ICU at the outside facility, but further decompensation and workup led to his transfer to our institution. His transfer was to manage a new left posterior inferior cerebellar artery (PICA) infarct and surgical evaluation of tricuspid valve (TV) and aortic valve (AV) endocarditis.

**Figure 1:**
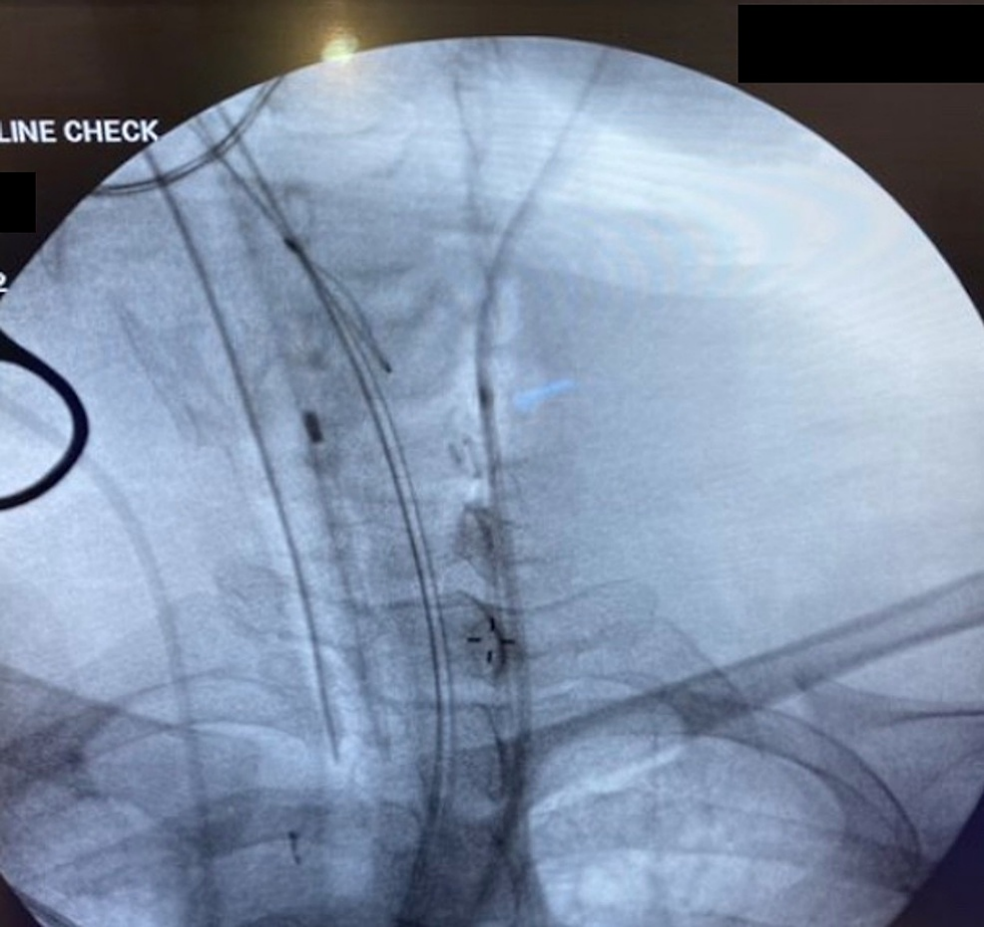
Anteroposterior X-ray Line Check

On the patient’s second day at our institution, a central line was placed in the ICU due to prolonged pressor requirements for septic shock. The procedure was completed under the normal standard of care and presumably placed in the IJV. However, waveform analysis suggested arterial cannulation, and vascular surgery was consulted. Vascular surgery workup suspected that the CVC had been placed in the carotid, but there was uncertainty related to common carotid versus internal carotid artery cannulation. An X-ray was obtained in an attempt to identify the cannulated vessel, but this was not definitive (Figure 1). Confirmation of arterial placement was completed with arterial blood gas (ABG) and central venous pressure (CVP) measurements. At this time, the patient was taken to the operating room (OR) for safe removal of the central line. 

**Figure 2:**
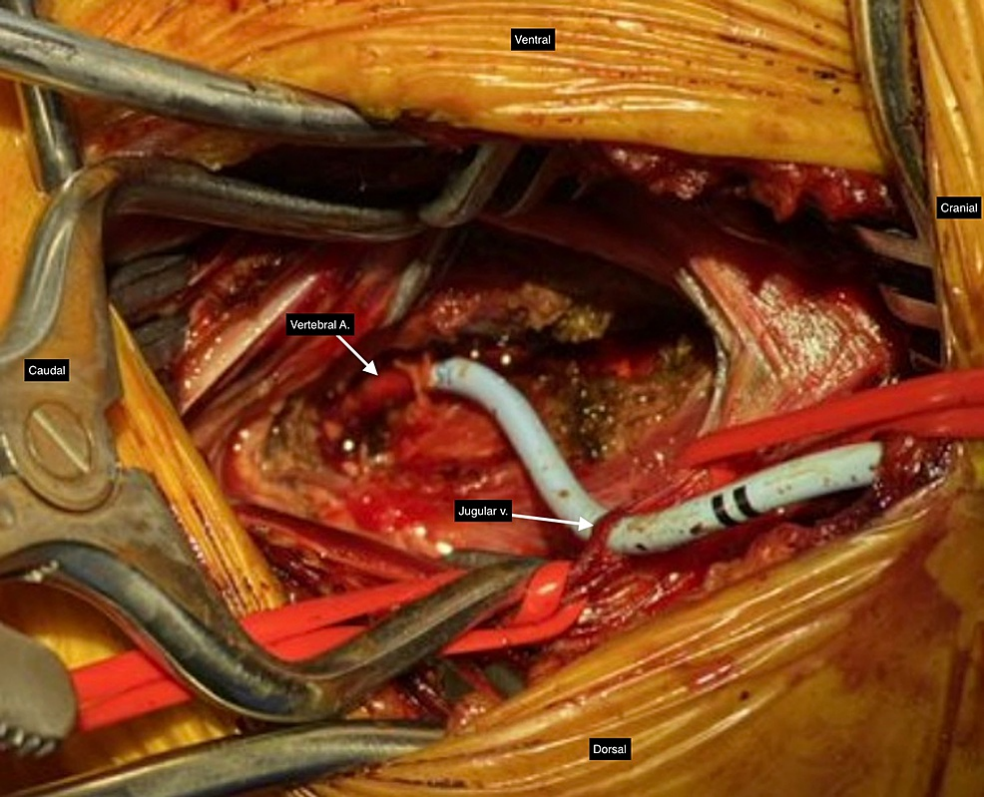
Primary Exposure of the Catheter Path

Once in the OR, initial dissection by the vascular surgery team revealed the CVC had been placed through and through the left internal jugular vein (Figure 2). However, with further dissection, it was determined the catheter was traveling lateral and deep to the carotid artery. At this time, the distal extent of the catheter was suspected to be in the vertebral artery and neurosurgery was consulted. Placement of the catheter in the vertebral artery was confirmed with contrast flush.

**Figure 3:**
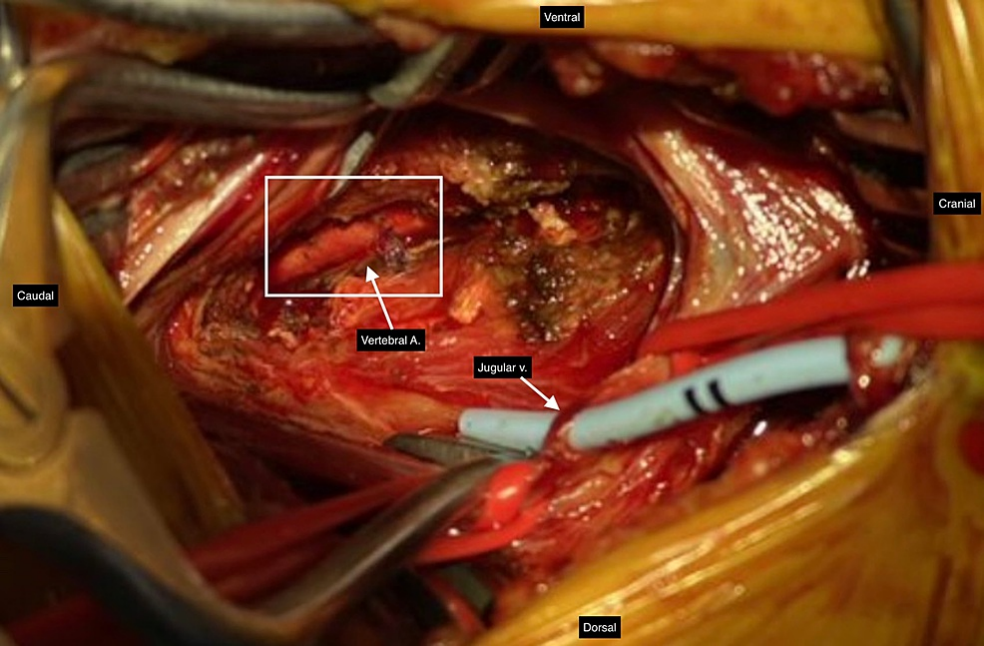
Primary Vertebral Artery Repair

Neurosurgery utilized a coarse diamond bit for resection of the transverse processes of the vertebral bodies bordering the cannulation site of the catheter, in the V2 segment of the vertebral artery. Following drilling, the bony exposure was completed with a rongeur, and the venous plexus oozing was controlled with bipolar cautery. Next, proximal and distal control of the vertebral artery was obtained. An aneurysm clip was placed distally, the catheter was removed, and another aneurysm clip was placed on the proximal end of the artery. Three interrupted 7-0 Prolene sutures were used to repair the vertebral artery (Figure 3). Following repair, multiphasic doppler signals were present proximal and distal to the repair site. The patient was then transferred back to the ICU.

## Discussion

The complications of central venous catheters should be at the forefront of the provider’s mind when completing this common procedure. As detailed by Kusminsky et al., complications occurring secondary to insertion can include arterial injury [7]. The risks of arterial injury can include hematoma, pseudoaneurysm formation, fistula formation, hemorrhage, and stroke secondary to occlusion of the artery [8]. Repair techniques of the vertebral artery following errant CVC placement have largely been described through case reports and case series. There is no standard method or approach to manage this complication. 

Typically, management of arterial injury during CVC placement can be managed in one of three ways: (1) removal of the catheter and direct pressure, (2) endovascular treatment, or (3) open surgical repair. The repair of the vertebral artery following cannulation by CVC is dependent upon the rapid identification and diagnosis of the complication. At present, the endovascular approach has been described in several case reports. As of 2014, Akkan et al. reported preservation of the vertebral artery via endovascular placement of a vertebral artery stent [9]. They also identify 46 prior cases of vertebral artery catheterization during CVC placement, but only three cases of successful parent artery preservation with endovascular stent placement [8-12]. Most recently, Rayes et al. presented a case in which they utilized an endovascular stent for vertebral artery preservation following iatrogenic injury [13]. Other endovascular treatments include embolization via either balloon or coil methods [14-17].

When endovascular methods are not conducive to repair, open techniques can be used. Prior reports have detailed excision of pseudoaneurysms and ligation of arteriovenous fistulas [18,19]. Given the typically delayed onset of symptoms associated with iatrogenic VA injury and the deep positioning of the VA, it is less amenable to open surgical repair. Despite this, open repair is the most reported strategy in the literature. The exposure provided by neurosurgery through the removal of the transverse processes of the C4 and C5 cervical vertebrae allowed visualization and control of the vessel to permit primary open repair. In addition, the rapid identification of arterial injury and lack of extravasation, hemorrhage, or other symptoms associated with the errant placement left the artery in a condition that was amenable to a primary repair.

The ability to repair the vertebral artery in a primary, open fashion will be limited and need to be evaluated on a case-by-case basis. In our situation, the intra-operative identification and small vessel puncture resulted in a situation that was amenable to exposure and repair. The deep nature of the vertebral artery limits its ability to be compressed, and the V2 segment running between transverse foramina will further complicate this strategy. However, the ability to approach this injury in a multi-disciplinary method for primary exposure via neurosurgery and the repair by vascular surgery led to an efficient, coordinated approach for repair. 

## Conclusions

The placement of central venous catheters will continue to be a frequently completed procedure given their utility. Knowledge of the pertinent complications is important in order to rapidly identify and effectively treat these patients. The vascular complications are especially important as the carotid, subclavian, and vertebral arteries are all present in the placement of CVCs. As we have shown, a multi-disciplinary approach for complete exposure and primary repair is a feasible and safe method to repair inadvertent vertebral artery injury in the V2 segment.
